# Sputum and nasal lavage lung-specific biomarkers before and after smoking cessation

**DOI:** 10.1186/1471-2466-11-35

**Published:** 2011-06-02

**Authors:** Izolde Bouloukaki, Ioanna G Tsiligianni, Maria Tsoumakidou, Ioanna Mitrouska, Emmanuel P Prokopakis, Irene Mavroudi, Nikolaos M Siafakas, Nikolaos Tzanakis

**Affiliations:** 1 Department of Thoracic Medicine, Faculty of Medicine, University of Crete, Crete, Greece; 2 Department of Epidemiology, Faculty of Medicine, University of Crete, Crete, Greece; 3 Department of Critical Care Medicine and Pulmonary Services, Evangelismos General Hospital, Athens, Greece; 4 Department of Otolaryngology, Faculty of Medicine, University of Crete, Crete, Greece; 5 Department of Hematology, Faculty of Medicine, University of Crete, Crete, Greece

## Abstract

**Background:**

Little is known about the effect of smoking cessation on airway inflammation. Secretory Leukocyte Protease Inhibitor (SLPI), Clara Cell protein 16 (CC16), elafin and human defensin beta-2 (HBD-2) protect human airways against inflammation and oxidative stress. In this longitudinal study we aimed to investigate changes in sputum and nasal lavage SLPI, CC16, elafin and HBD-2 levels in healthy smokers after 6 and 12 months of smoking cessation.

**Methods:**

Induced sputum and nasal lavage was obtained from healthy current smokers (n = 76) before smoking cessation, after 6 months of smoking cessation (n = 29), after 1 year of smoking cessation (n = 22) and from 10 healthy never smokers. SLPI, CC16, elafin and HBD-2 levels were measured in sputum and nasal lavage supernatants by commercially available ELISA kits.

**Results:**

Sputum SLPI and CC-16 levels were increased in healthy smokers before smoking cessation versus never-smokers (p = 0.005 and p = 0.08 respectively). SLPI and CC16 levels did not differ before and 6 months after smoking cessation (p = 0.118 and p = 0.543 respectively), neither before and 1 year after smoking cessation (p = 0.363 and p = 0.470 respectively). Nasal lavage SLPI was decreased 12 months after smoking cessation (p = 0.033). Nasal lavage elafin levels were increased in healthy smokers before smoking cessation versus never-smokers (p = 0.007), but there were no changes 6 months and 1 year after smoking cessation.

**Conclusions:**

Only nasal lavage SLPI decrease after 1 year after smoking cessation. We may speculate that there is an ongoing inflammatory process stimulating the production of counter-regulating proteins in the airways of healthy ex-smokers.

## Background

Smoking exerts its primary effects on the respiratory tract, and is associated with the development of several diseases such as cancer and chronic obstructive lung disease (COPD) [1]. COPD involves airway inflammation and a probable protease-antiprotease imbalance. However, little is known about the underlying mechanisms and how they are related to the susceptibility to the disease, particularly why only 10-15% of smokers develop COPD [2]. Currently, there are no biological markers sufficiently sensitive to assess parenchymal lung health before functional abnormalities become apparent.

Consequently, lung-specific biomarkers, indicating epithelial cell activation or damage, produced by distinct regions of the respiratory tract, are desirable for evaluation of smoking exposure, effects and identification of susceptible individuals to smoking. The respiratory epithelium, according to anatomical location (nose, trachea, bronchi, distal airways and alveoli), secretes several specific proteins into the airspaces of the respiratory tract. These include Secretory Leukocyte Protease Inhibitor (SLPI), the 16-kDa bronchiolar Clara cell secretory protein (CC16), elafin, as well as human beta β-defensins-2 (hBD-2).

Secretory Leukocyte Protease Inhibitor (SLPI) and elafin, two serine protease inhibitors, are produced by a number of cell types in the respiratory tract. They protect local tissue against the detrimental consequences of inflammation not only as a result of their anti-inflammatory activities but also via their antiprotease and antimicrobial properties [3,4]. CC16 is a sensitive marker of cigarette smoking exposure and one of the main secretory proteins in the lung [5]. CC16 is produced mainly by Clara cells in the lung and also by non-ciliated cells along the tracheobronchial epithelium and similar cell types in the nasal cavity [6]. It plays an important protective role in the respiratory tract against oxidative stress and inflammation [7]. HBD-2, widely expressed in epithelial cells lining the respiratory tract, is primarily known for its antimicrobial activities [8]. However, it is now clear that it has anti-inflammatory effects as well [9].

Serum, sputum, Bronchoalveolar Lavage Fluid (BALF), nasal lavage and bronchial epithelial cell expression of CC16 is reported to be lower in smokers with normal lung function in most of the studies [5,10-18]. These changes seem to be related to pathogenesis of smoke-related lung diseases. We and others have found a further decrease in CC16 in COPD and its exacerbations [11,14,19,20]. However, in one study, healthy smokers had a relative high level of CC16 in the airways, with similar levels in bronchial fluids between smokers and non-smokers [21]. Furthermore, we have found increased levels of CC16 in sputum and Lindahl et al found higher levels of CC16 in BALF and nasal lavage of smokers than nonsmokers, which is in contrast with other previous reports [6,20].

Currently little is known about the effects of smoking on the other biomarkers. Previously we have found increased SLPI in sputum in healthy smokers compared to never-smokers, which is further increased in COPD patients, but no difference in elafin and HBD-2 between the two groups [20]. Former studies have shown that acute exposure to cigarette smoke in the mouse lung induces a loss of activity of SLPI [22], while chronic smoke exposure increased SLPI expression in the smoke-exposed mice [23]. Moreover, smoke exposure has been associated with significantly reduced HBD-2 levels in pharyngeal washes and sputum of patients with acute pneumonia, suggesting increased susceptibility to infection [24]. This mechanism may be important in the pathogenesis of COPD, as increased defensin levels are observed in inflammatory lung diseases [25]. To the best of our knowledge there are no studies about the effect of smoking on elafin levels.

It remains uncertain whether expression of these biomarkers is altered after smoking cessation. There is only one longitudinal study in BALF, which showed significant higher levels of CC16 after smoking cessation, which failed to remain after 15 months of smoking cessation [26]. No other longitudinal studies have reported the effect of smoking cessation on SLPI, elafin and HBD-2 in "healthy" smokers. We have conducted a longitudinal study to examine whether changes in sputum and nasal lavage SLPI, CC16, elafin and HBD-2 are reversed after a period of 6 months and 12 months smoking cessation in "healthy" smokers.

## Methods

### Subjects

In the University Hospital of Heraklion, Crete induced sputum and nasal lavage was obtained from 76 smokers, recruited from the smoking cessation clinic, before their entrance into the smoking cessation protocol. All subjects provided written informed consent and ethical approval was provided by the University Hospitals Ethics Committee. Following enrollment, all smokers participated in the same behavioural therapy program and according to their degree of dependence (Fagerstrom test) they received nicotine replacement therapy and/or bupropion as part of their smoking cessation treatment. Ten never-smokers were recruited by advertisement and were the control subjects. All subjects were free of any respiratory tract infections at the time of the study or during the month preceding the study and at the follow-up at 6 and 12 months. Subjects did not suffer from any disease and did not receive any medication at the time of the first visit, and none had symptoms of chronic bronchitis. Furthermore, baseline lung functional tests were within the normal range and their chest radiographs were normal. During each follow-up, confirmation of smoking status was assessed by exhaled CO.

The participating subjects' characteristics are depicted in Table 1. The studied population participated in a parallel study aiming to investigate the levels of the same biomarkers in COPD and IPF. Results from that study have been recently published [20].

**Table 1:** Subject characteristics

Time	Smokers			Never-smokers
				
	*Before sm-ces 0*	*After sm-ces 6 m*	*After sm-ces 12 m*	0
**N**	76	29	22	10
**Sex, M/F**	65/11	26/3	20/2	8/2
**Age, (yr)**	48 ± 9	48 ± 8	47 ± 7	33 ± 9*
**Smoking (p-yr)**	57 ± 18	53 ± 16	56 ± 18	N/A
**FEV_1_,%pred**	104 ± 11	105 ± 13	108 ± 9	114 ± 14
**FEV_1_/FVC%**	80 ± 6	81 ± 5	80 ± 6	87 ± 6
**Fagerstrom score**	8 ± 2	7 ± 1	7 ± 1	N/A

Data are presented as mean ± SD, or in absolute numbers. sm-ces: smoking cessation, N: numbers, M: male, F: female, yr: years, p-yr: pack years, FEV_1_: forced expiratory volume in one second, FVC: forced vital capacity, N/A: non applicable

* p < 0.05 versus successful quitters at baseline,

### Sputum induction and processing

Sputum was induced and processed as previously described [27]. Subjects inhaled hypertonic saline for three ten-minute sessions. The concentration of the inhaled saline was consecutively increased in each session from 3%, to 4% and to 5%. Always between sessions the inhalation procedure was interrupted and the subjects were asked to blow their nose, rinse their mouth and try to expectorate sputum into a sterilized box. By this way saliva contamination of the sample was minimized and the percentage of squamous cells in the sample was decreased. Sputum was processed within the next 30 min or no more than two hours, with the sample always kept in ice. The volume of the sputum was measured and sputum samples with a volume of at least 2 mL are reputed to be sufficient. The more viscid proportions of the sputum (plugs) were selected and weighed. Dithiothreitol (Calbiochem, Darmstadt, Germany) was added, followed by phosphate buffer saline (PBS). Then, the mixture was filtered and centrifuged. The sol phase was removed and stored at -80°C until analyzed. The cell pellet was resuspended with PBS, a total cell count of the sample was performed and viability was tested by means of trypan blue exclusion method. The sample was considered for further analysis if squamous cell contamination was < 20% and cell viability was > 50%,. Sputum cytospin slides were stained using May-Grunwald Giemsa for differential cell count. Cell counting was performed by one investigator (IB), blind to the origin of the samples. At least 400 cells were counted. Cytospins with < 50% squamous cells and > 400 nonsquamous cells were qualified as of good quality. Cell differential counts were expressed as % of total sputum non-squamous cells.

### Nasal lavage

Briefly, the subjects, in a seated position, were instructed to flex their neck approximately 30° from the vertical and to not breathe through their nose. Five milliliters of normal saline was gently instilled into each nostril using a syringe with a dwelling time of approximately 10 seconds. The lavage fluid was then collected by repeated aspiration from both nasal cavities, kept on ice, weighed, and processed within 1 hour (centrifugation for 10 minutes at 400 g and storage of supernatant at -80°C pending analysis).

### Assessment of mediators

Commercially available ELISA kits were used to measure SLPI (R&D Systems Europe Ltd, Abingdon, UK), CC16 (Biovendor GmbH, Heidelberg, Germany), elafin (R&D Systems Europe Ltd, Abingdon, UK), and HBD-2 (EMELCA Bioscience, Breda, The Netherlands), in accordance with the manufactors instruction. "Recovery" experiments were performed to determine if the kits' reagents were suitable for application in nasal lavage and sputum samples. Samples were split into two aliquots and one aliquot was run as "neat or unspiked", while the other aliquot was "spiked" with a small volume of the kit standard concentrate, then compared to a "spiked" diluent. The spiked sample was also tested by diluting it to determine if it dilutes parallel to the standard curve. The recoveries were always in the range of 80-120%.

Assays were performed in duplicate. The readings of each standard and sample were averaged and the average of zerostandard was subtracted The mean intra-sample CV was < 4%. The detection limits of SLPI, CC16, elafin and HBD-2 were 62.5 pg/ml, 20 pg/ml, 31.25 pg/ml and 8 pg/ml respectively.

### 2.3. Statistical Analysis

Normality was tested by the Shapiro-Wilk test. Variables were non-normally distributed. Only successful quitters at 6 months (n = 29) and 1 year (n = 22) were included in the analysis of repeated measurements using the Friedman test followed by the Wilcoxon Signed Ranks test to see pair-wise differences. Differences between smokers and the control group were tested using the Mann-Whitney U test. The software StatsDirect (SPSS Statistics 17.0.0 CHICAGO IL, USA) was used. Probability values of < 0.05 were considered as statistically significant.

## Results

A total of 76 smokers and 10 controls enrolled in the study. Successful quitters were 29/76 (38.2%) at 6 months and 22/76 (29%) at 1 year. Good quality sputum specimens were obtained a) at baseline from 68 "healthy" current smoker subjects (8 subjects declined to have sputum induction, b) after 6 months of smoking cessation from 21 of 29 subjects who successfully managed to quit smoking, c) after 1 year of smoking cessation from 14 of 22 who had not relapsed, and d) from all 10 controls. Nasal lavage was obtained a) at baseline from 69 "healthy" current smoker subjects (7 subjects declined to have nasal lavage) b) after 6 months of smoking cessation from 22 of 29 subjects who successfully managed to quit smoking, c) after 1 year of smoking cessation from 12 of 22 who had not relapsed, and d) from all 10 controls.

Forty of all the participants received bupropion (53%), 5 nicotine replacement therapy (7%), 26 both agents (34%) and 5 refused to receive any treatment (7%). The duration of treatment was median (range), 4 (0-10) weeks. Of those who successfully quitted smoking at 6 months (n = 29), 19 received bupropion (66%), 1 nicotine replacement therapy (3%), 8 both agents (28%) and 1 refused to receive any treatment (3%), while of those who successfully quitted smoking at 1 year (n = 22), 16 (72%) received bupropion, 1 nicotine replacement therapy (5%) and 5 both agents (23%). The duration of treatment was 5 (0-8) weeks for successful quitters at 6 months and 5 (0-8) weeks for successful quitters at 1 year.

Concentrations of SLPI, CC16, elafin and HBD-2 in sputum and nasal lavage are shown in Table 2 and Table 3, respectively. The concentration of SLPI and elafin was significantly higher in nasal lavage than in the sputum (p = 0.0001 and p = 0.022, respectively). The concentration of CC-16 was higher in sputum compared to nasal lavage (p = 0.001). However, the levels in most of the nasal lavage samples of HBD-2 were below the detection limit so we did not report those results.

**Table 2:** Concentrations of measured variables in sputum.

	Smokers			Controls (n = 10)
	**Before** **(n = 21)**	**6 m sm cessation** **(n = 21)**	**12 m sm cessation** **(n = 14)**	

**SLPI** **(μg/ml)**	1.11 (0.3-4.9)	0.8 (0.2-2.6)	1.36 (0.3-3.3)	0.1 (0.05-0.6)*

**CC16** **(ng/ml)**	1700 (300-13000)	1800 (100-21000)	1900 (60-5500)	500 (1-4800)*

**b-defensin** **(pg/ml)**	74.26 (8 - 534,91)	98.1 (10.2 - 729.9)	83.1 (8 - 256)	73.5 (34.6 - 409.9)

**Elafin** **(pg/ml)**	252.9 (92.8 - 2146.7)	171.9 (71.2 - 472.9)*	236.4 (44.6 - 714.1)	194.6 (128.8 - 276.8)

Data are presented as median (range). Sm cessation: smoking cessation.

* p < 0.05 versus successful quitters at baseline

**Table 3:** Concentrations of measured variables in nasal lavage fluid.

	Smokers			Controls (n = 10)
	**Before** **(n = 22)**	**6 m sm cessation (n = 22)**	**12 m sm cessation (n = 11)**	

**SLPI (μg/ml)**	3.2 (0.1-37.5)	1.96 (0.2-25.8)	1.9 (0.05-13.4)*	2.7 (0.2-7)

**CC16 (ng/ml)**	26.3 (1.5-200)	34 (0.8-169.4)	38.9 (0.8-80)	25.6 (1.3-200)

**b-defensin (pg/ml)**	-	-	-	-

**Elafin (pg/ml)**	6701.5 (1195.8-17360.5)	5927.8 (339.5-13298.4)	3284 (328.9-7398)	1665.5 (484.9-5894.2)*

Data are presented as median (range). Sm cessation: smoking cessation.

* p < 0.05 versus successful quitters at baseline

In sputum, SLPI and CC16 levels were increased in healthy smokers before smoking cessation versus never-smokers (p = 0.005 and p = 0.08 respectively). However, SLPI and CC16 levels did not differ before and 6 months after smoking cessation (p = 0.118 and p = 0.543 respectively), and neither before and 1 year after smoking cessation (p = 0.363 and p = 0.470 respectively). Furthermore HBD-2 and elafin levels did not differ in healthy smokers before smoking cessation versus never-smokers. HBD-2 did not differ before and 6 months and 1 year after smoking cessation. Although elafin was lower 6 months after smoking cessation (p = 0.025), 12 months after smoking cessation its levels were the same as those before smoking cessation. Moreover, there was no difference in nasal lavage, SLPI, CC16 between healthy smokers before smoking cessation and never-smokers. SLPI was lower 12 months after smoking cessation (p = 0.033). Nasal lavage elafin levels were increased in healthy smokers before smoking cessation versus never-smokers, p = 0.007. However, there were no changes in nasal lavage elafin and CC16 6 months and 1 year after smoking cessation. We did not find any correlation in mediator concentrations between each other.

## Discussion

The present study was performed to examine respiratory fluid obtained from the nose and induced sputum for lung-specific biomarkers. BALF has been used to assess airway inflammation in various diseases, but as it is a difficult invasive method and has several limitations it can not be used extensively. Sputum induction has been proposed as a non-invasive method that could give valuable information for lung injury, the Th1/Th2 response, the inflammatory process, and the effects of pharmacological treatment [28]. Our primary endpoint was to detect tobacco insults to the respiratory epithelium and study the signs of possible repair after smoking cessation. We chose nasal lavage fluid and sputum because they are easy, non-invasive methods that additionally contain a large number of proteins, which comprise a potential source for detecting and characterizing biochemical alterations associated with airway diseases. It may also be possible to monitor protein changes but also give information of inflammation processes in the lower airways [27,29]. Biomarkers of oxidative stress and airway inflammation including 8-isoprostane [30] and leukotriene B4 [31] have also been measured in exhaled breath condensate, a non-invasive method for sampling airway secretions [32], in healthy smokers and patients with COPD. Furthermore, electronic nose is a promising non-invasive technique for assessment of airway inflammation [33].

SLPI and elafin are elastase inhibitors that seem to play an important role in protease-antiprotease balance and in controlling elastase levels. Additionally, this investigation could be considered extremely important in diseases such as COPD and asthma where the cytokine network plays a key role and therefore could potentially be used in the future as therapeutic agents. We chose SLPI, CC16, elafin and HBD-2 because they are key proteins that have been found in human airway secretions indicating respiratory epithelial cell activation or damage [3-8] for evaluation of smoking exposure and signs of possible repair after smoking cessation. Regarding the validation of the mediators, our study confirmed that the assay for the 4 mediators were all reproducible with each of these assays giving > 80% recovery of a "spike" with pure reagent.

Our results showed that sputum SLPI, CC16, elafin and HBD-2 levels do not decrease up to 1 year after smoking cessation. We can not exclude the possibility that a decrease could take place the following years [26] as our study was limited to 12 months. The Clara Cell Secretory protein after 15 months of smoking cessation declined to the reduced levels noted before smoking cessation. Twelve months of smoking cessation decrease significantly only nasal lavage SLPI, suggesting that there is an ongoing inflammatory process stimulating the production of counter-regulating proteins in the airways of healthy ex-smokers. The differences found between sputum and nasal lavage need further investigation as contrary to that expected only nasal lavage showed a decrease in the investigated levels.

Reports on serum, sputum, nasal lavage, BALF and bronchial epitheliums CC16 levels are controversial, showing either a decrease [5,10-18], no change [21] or an increase [6] in smokers with normal lung function compared to never smokers. These discrepancies could reflect the difficulty in comparing CC16 in the fluid phase of respiratory samples, mainly for methodology reasons. Also it is difficult to compare published studies in serum with others in sputum and nasal lavage as firsts are representative of the entire body rather than only the lung burden. We found higher levels of CC16 in sputum of smokers compared to nonsmokers, which is in accordance with the study of Lindahl et al [6]. It seems likely that the increase in CC16 levels observed in smokers in the present study reflects a regenerative process involving the Clara cells. The higher levels could also explain why these subjects did not develop COPD as in COPD usually a decrease is observed [14]. Further longitudinal studies are needed in order to clarify which are the potential other factors that make some smokers as in our study to have high levels of CC16 contrary to the other previous studies and which is exactly the role of these mediators. Probably an earlier step in the immune response or other associated factors are responsible of either increase, decrease or no change. Further investigation is needed and should considered important as it could in part explain the reasons of why some smokers develop COPD and some do not. Furthermore the increase in sputum SLPI and nasal lavage elafin in smokers might be a part of the local defence against inflammatory processes to minimize tissue damage induced by smoking. SLPI and elafin are characterized as alarm molecules involved in the regulation of early events in the inflammatory process [34], therefore the overexpression of these inhibitors would be of benefit in combating the inflammatory consequences of smoking. The possible important role of these two anti-inflammatory proteins has also been shown in a recently published study of our group where elafin was increased only in smokers that develop COPD. On the other hand SLPI is increased in smokers independently of whether they have developed COPD or not [20]. This has also been confirmed in our study where smokers with no lung disease have increased SLPI.

We did not find any changes in sputum HBD-2 between smokers and non-smokers and there are not enough data in the literature about the effects of smoking on HBD-2, recruiting healthy smokers. Smoking has been proven to be a factor for bacterial colonisation in the respiratory tract. Toll-like receptors (TLRs) possess a key role in host defence in the respiratory tract. The TLR4 stimulation results in production of HBD-2, a part of the innate host defence. In a study that used surgical specimens from current smoker COPDs, former smoker COPDs, healthy ex-smoker and smoker subjects the expression of HBD2 in the epithelium of distal airways was higher only in current smokers with COPD [35]. MacRedmond et al showed TLR2 expression was unchanged in the nasal epithelium of smokers compared with non-smoking controls [36]. There is only one study in patients with acute pneumonia, reporting reduced HBD-2 levels in pharyngeal washes and sputum in current and former smokers [24].

To the best of our knowledge there is very limited evidence that has studied the alteration of these biomarkers after smoking cessation. The present study is one of the first longitudinal studies that investigate the effect of smoking cessation on SLPI, CC-16, elafin and HBD-2 in induced sputum and nasal lavage of smokers with no concurrent disease or lung function impairment.

One previous longitudinal study assessing the effect of 1, 3,6, 9 and 15 months smoking cessation in smokers with normal lung function, showed significantly (p < 0.05) higher levels of CC-16 in BAL fluid at 3, 6, and 9 months after smoking cessation. However 15 months after smoking cessation CC-16 levels were the same as those before smoking cessation [26]. In accordance with this study we found that sputum and nasal lavage CC-16.levels do not change up to 1 year of smoking cessation. This could be explained by an irreversible insult to the bronchiolar epithelium from tobacco smoke, suggesting that the regenerative process is incomplete. Another explanation is that more than 12 months of smoking cessation are needed to observe permanent changes in lung biomarkers related to cigarette smoking.

Our understanding of the effect of smoking and smoking cessation on HBD-2 and elafin is limited at present and this should be an area of study in the coming years. Future studies are required to elucidate their role in the pathogenesis of inflammatory lung diseases [25].

### Limitations-Strengths

Our study has some limitations that deserve comment. Firstly, our data could be criticised by the fact that 29 and 22 individuals (successful quitters at 6 months and 1 year, respectively) completed the evaluation. However this is common in smoking cessation clinics where relapse is frequent. Further we restricted our repeated analysis only in successful quitters. Moreover, our control group included only 10 subjects, which did not match with the number of healthy smokers, similar with other studies using induced sputum attempt. Although, there is a concern about the possibility of statistical error type-II, the comparisons between healthy smokers and non-smoking control individuals are not susceptible for this type error, because we have already detected statistical significance. Statistical type II is possible in the case of SLPI measurements where we detected a tendency of important but statistically insignificant differences and larger studies are needed to verify the issue. Thus the power of the study maybe improved, in the case of SLPI, only by increasing the number of healthy smokers, successful quitters, reaching the appropriate end-study sample size of 170 individuals. Taking into account that in cessation clinics worldwide only 25-30% are successful quitters in the first year and that the success of sufficient sputum is 80% we need to recruit 800 individuals at baseline to obtain adequate power. Secondly, nasal lavage samples the nose and sputum induction samples primarily the lumen of the central airways. Consequently, sputum and nasal lavage changes in lung-specific biomarkers do not necessarily reflect changes in airway wall. However, common methods to study tissue, such as the examination of bronchial biopsies or surgical specimens cannot be easily applied on longitudinal studies. Thirdly, the mean age of the smokers' group was greater than that of the control group and it could be argued that differences between smokers and never-smokers are due to the age difference. Although this cannot be excluded, an analysis of subgroups of different ages did not show differences (data not shown). Furthermore, as sputum was treated with the mucolytic agent dithiothreitol (DTT), a possible influence of DTT treatment on detection of these soluble mediators has to be considered. The reducing agent DTT could have interfered with the detection of inflammatory mediators in the sputum sol phase, either by affecting the three dimensional structure of proteins causing the release of mucus bound molecules, or by interfering directly with the immunoassay. In the present study a detailed analysis of the effects of DTT on these was not performed. However, several studies have excluded that DTT has an effect on mediators. Woolhouse et al, has investigated the effect of sputum processing with DTT on detection of inflammatory mediators and showed that DTT does no significantly affect median levels of SLPI [37]. Moreover, de Burbure et al assessed the effect of DTT treatment on the concentrations of CC16 by adding DTT to sputum samples. Concentrations of CC16 in treated and untreated samples were highly correlated with no systematic difference (post-treatment values averaged 102% of the pre-treatment values) [38].

## Conclusions

In conclusion, we have shown that sputum biomarkers levels do not decrease up to 1 year after smoking cessation. Twelve months of smoking cessation decrease significantly only nasal lavage SLPI, suggesting that there is an ongoing inflammatory process stimulating the production of counter-regulating proteins in the airways of healthy ex-smokers. Further investigations are needed to explore if these proteins could be valid biomarkers for monitoring peripheral airway damage.

## List of abbreviations

BALF: Bronchoalveolar Lavage Fluid; CC16: Clara Cell protein 16; COPD: Chronic Obstructive Pulmonary Disease; HBD-2: human defensin beta-2; SLPI: Secretory Leukocyte Protease Inhibitor; TLRs: Toll-like receptors.

## Competing interests

The authors declare that they have no competing interests.

## Authors' contributions

IB, MT, NT and NS conceived the hypothesis and designed the study. IB (foremost), EP, IM performed the experiments. All authors have participated in different steps of the procedures, contributed to interpretation of data, writing the article and revising it critically for important intellectual content and final approval of the version to be published. All authors read and approved the final manuscript.

## Pre-publication history

The pre-publication history for this paper can be accessed here:

http://www.biomedcentral.com/1471-2466/11/35/prepub
